# Genotype, risk, and the future of precision medicine in dilated cardiomyopathy

**DOI:** 10.1093/ehjvshd/xwag053

**Published:** 2026-10-08

**Authors:** Prateeti Khazanie, Matthew R G Taylor, Luisa Mestroni

**Affiliations:** Division of Cardiology, University of Colorado Anschutz Medical Campus, 12505 E. 16th Ave, Anschutz Inpatient Pavilion 2, Aurora, CO 80045, USA; Division of Cardiology, University of Colorado Anschutz Medical Campus, 12505 E. 16th Ave, Anschutz Inpatient Pavilion 2, Aurora, CO 80045, USA; Adult Medical Genetics Program, Department of Medicine, University of Colorado Anschutz Medical Campus, 12700 E 19th Ave # F442, Aurora, CO 80045-2507, USA; Division of Cardiology, University of Colorado Anschutz Medical Campus, 12505 E. 16th Ave, Anschutz Inpatient Pavilion 2, Aurora, CO 80045, USA

**Keywords:** Dilated cardiomyopathy, Genetic testing, Risk stratification, Precision medicine

Dilated cardiomyopathy (DCM) remains one of the most challenging diseases in cardiovascular medicine because it is inherently heterogeneous. Patients who appear clinically indistinguishable, with identical left ventricular ejection fraction (LVEF), comparable late gadolinium enhancement (LGE) on cardiac magnetic resonance imaging, and similar symptom burden, can follow strikingly divergent clinical courses. One develops malignant ventricular arrhythmia at a young age; another remains stable into the seventh or eighth decade. Such divergence reflects underlying biological heterogeneity that is only partially captured by conventional risk markers.

For decades, risk stratification in DCM has been anchored to a phenotype-first paradigm that asks: ‘how sick does this heart look?’. The answer, derived from LVEF, ventricular dimensions, myocardial fibrosis burden, and electrical arrhythmias, has long guided clinical decisions regarding medical management, implantable cardioverter-defibrillator (ICD) implantation, surveillance intensity, and referral for advanced heart failure (HF) therapies including heart transplantation or mechanical circulatory support. Although indispensable, these tools are insufficient. Identifying patients at risk for sudden cardiac death or progressive HF ultimately requires reframing the question: ‘what is this heart programmed to do?’.

Genetic testing has transformed our understanding of DCM pathobiology. Pathogenic or likely pathogenic variants have been identified in ∼19%–43% of unselected patients using contemporary sequencing panels, rising to over 50% in familial disease.^1–4^ Evidence from cohort studies has established that phenotype and prognosis vary substantially by genotype,^2–7^ yet few studies have provided a direct, head-to-head comparison of clinical outcomes across individual genetic subtypes. Many studies have relied on genotype clustering for statistical power, often at the expense of gene-specific insights.^7,8^

In this issue, Cannie and colleagues report a single-centre, retrospective longitudinal cohort study from the UCL/Barts Heart Centre that included 484 clinically affected patients carrying pathogenic or likely pathogenic variants in five major DCM-associated genes (*TTN*, *DSP*, *LMNA*, *RBM20*, and *FLNC*) and a genotype-negative reference cohort. Over a median follow-up of 48 months, 57 patients (11.8%) reached the composite endpoint of malignant ventricular arrhythmias (MVA) or end-stage HF. A clear, genotype-stratified risk hierarchy emerged. Genotype-negative patients had the lowest incidence of adverse events, and *TTN* cardiomyopathy conferred a similar risk profile. Rates increased stepwise: approximately two-fold higher in *DSP* and *FLNC*, three-fold in *RBM20*, and four-fold in *LMNA* cardiomyopathy. These findings reinforce the concept that DCM represents a collection of biologically distinct disease entities rather than a single syndrome.^9^ More important than the magnitude of the risk differences is their structure: the baseline predictors of outcome differed systematically across genotypes. LVEF was associated with the primary endpoint in genotype-negative, *DSP*, and *RBM20* patients, but not in *LMNA* or *TTN* disease. LGE on CMR predicted outcome in genotype-negative patients but not in *DSP* cardiomyopathy, where near-universal presence of LGE limits its discriminatory value. Right ventricular function, measured by tricuspid annular plane systolic excursion, was informative only in *DSP* disease. Together, these findings suggest that risk markers are genotype-specific rather than universal, challenging one-size-fits-all risk models.

Several limitations merit consideration. The cohort was derived from a tertiary referral centre and was enriched for severe and early-onset disease. Genotype-negative patients had the highest proportion of probands (94.4%), likely reflecting ascertainment bias towards more severe index cases. Sample sizes were modest for several genotypes, particularly for *FLNC* (*n* = 15 for survival analysis) and *RBM20* (*n* = 18), limiting statistical precision and genotype-specific multivariable analyses. Furthermore, the genotype-negative category was itself heterogeneous, reflecting current limitations in variant detection rather than the absence of genetic contribution. Future incorporation of polygenic risk and more comprehensive genomic approaches may further refine this population. These limitations underscore the need for large, harmonized, international multicentre registries capable of supporting genotype-specific risk models.

The observed risk hierarchy described by Cannie *et al*. reflects fundamental biological differences between DCM genotypes. Rather than representing variations of a single disease, these entities represent distinct endotypes with characteristic clinical trajectories and mechanisms of disease.

*LMNA* cardiomyopathy demonstrates the highest overall event rate in this cohort, with 35.9% of patients reaching the primary endpoint at 5 years. Its phenotype extends well beyond systolic dysfunction: 47.6% of *LMNA* patients developed atrial fibrillation during follow-up, 21.4% progressive conduction disease, and 19% ultimately required heart transplantation.^6^ Critically, MVA occurred in *LMNA* patients with LVEF ≥35%, illustrating the limitations of conventional LVEF thresholds for risk assessment in laminopathy. The validated LMNA arrhythmia risk score, incorporating non-sustained ventricular tachycardia, LGE, male sex, and LVEF as genotype-specific variables, provides an example of how genotype-specific risk models can refine risk stratification within a precision framework.^10^

*RBM20* cardiomyopathy presents with the youngest median age at clinical diagnosis (35 years) in this cohort and among the highest rates of appropriate ICD therapy (29.4%), confirming its status as an aggressively arrhythmogenic disease. Its end-stage HF burden at 5 years was comparatively low, unlike *LMNA* cardiomyopathy, though the small sample constrains firm conclusions. Prior work has characterized the *RBM20* phenotype in greater depth, demonstrating that hotspot variants in the arginine and serine-rich domain carry the most severe arrhythmic risk and further emphasizing the biological heterogeneity of DCM.^11^

*FLNC* truncating variant cardiomyopathy is characterized by extensive LGE (93.8%), a relatively preserved ejection fraction at presentation (median 48%), and a statistically elevated MVA risk despite a modest cohort size, whereas no end-stage HF events were observed during follow-up.^12^ This dissociation between arrhythmic and HF risk defines *FLNC* cardiomyopathy as a primary electrical disease with a fibrotic structural substrate, suggesting that surveillance and device strategies may need to differ from those used in other forms of DCM. A recently validated *FLNC*-specific arrhythmic risk stratification tool, incorporating genotype-specific CMR and arrhythmia variables, further supports this concept.^13^

*DSP* cardiomyopathy occupies an intermediate risk position (Incidence Rate Ratio or IRR 2.0) but carries a phenotypic signature that is distinctive and mechanistically informative. The highest premature ventricular contraction burden of any genotype studied (median 1700/24 h), near-universal LGE, and a 10.9% rate of clinically suspected myocarditis during follow-up are consistent with an inflammatory and fibrotic pathophysiology that differs fundamentally from the mechanisms underlying other high-risk genotypes.^14^ The paper appropriately highlights that *DSP* with predominant left ventricular involvement may represent a lower-risk disease than *LMNA*, *RBM20*, or *FLNC*, with implications for how *DSP* cardiomyopathy is classified and managed at the intersection of DCM and arrhythmogenic cardiomyopathy.^15^

*TTN* cardiomyopathy, the most common genetic form of DCM, carries a composite risk indistinguishable from genotype-negative patients in this cohort, consistent with previous reports suggesting a more treatment-responsive phenotype.^5^ These findings should not be interpreted as evidence of benign disease. Nearly one in five *TTN* patients had an HF admission, atrial fibrillation was common, and female *TTN* carriers had the highest rates of peripartum cardiomyopathy, with implications for reproductive counselling and postpartum surveillance. *TTN* cardiomyopathy demands vigilant monitoring, prompt optimization of guideline-directed medical therapy, and family screening; its more favourable arrhythmic and end-stage HF risk profile simply means that device decisions should be guided by conventional criteria rather than genotype alone. Taken together, these data underscore that genotype reflects disease mechanism rather than functioning as a simple risk marker. Increasingly, DCM appears less like a single disease and more like a family of endotypes with distinct trajectories, mechanisms, and risk profiles.

Risk stratification is only valuable insofar as it improves outcomes. The genotype-stratified risk framework established by Cannie and colleagues is clinically actionable because it informs decisions that alter disease trajectory and signals a transition towards genotype-directed therapy. For the highest-risk genotypes, the case for early and proactive device implantation is now reinforced by present data. ICD consideration in *LMNA*, *RBM20*, and *FLNC* cardiomyopathy should not be deferred until LVEF falls below 35%, given the documented occurrence of MVA at preserved ejection fractions across all three groups. For *LMNA* disease specifically, the arrhythmic risk score provides the type of genotype-specific framework that is increasingly incorporated into contemporary practice guidelines.^10,16^ Likewise, the inflammatory ‘hot phase’ phenotype of *DSP* cardiomyopathy raises the possibility that targeted anti-inflammatory approaches warrant further investigation.^14^ Beyond device therapy, molecular and pharmacological strategies are increasingly being tailored to disease biology.^17^ Examples of emerging genotype-directed therapies are summarized in *Table 1*. Across genotypes, cross-cutting therapeutic strategies are also emerging. Phospholamban-targeting antisense oligonucleotides have demonstrated potential benefit across genetically heterogeneous DCM, including *TTN*, *LMNA*, *RBM20*, *MYH7*, and *TNNT2*, by modulating a shared downstream pathway of calcium dysregulation, suggesting that phospholamban (PLN)-axis intervention may offer benefit independent of the upstream genetic cause.

**Table 1:** From genotype to precision therapy in dilated cardiomyopathy

Genotype	Biology	Phenotype	Risk profile	Emerging therapeutic opportunities
*LMNA*	Nuclear envelope dysfunction	Arrhythmias, conduction disease, HF progression	Highest risk: arrhythmic and HF	MEK inhibition, genotype-specific ICD strategies, gene therapy
*RBM20*	Aberrant RNA splicing	Early-onset arrhythmogenic DCM	High arrhythmic risk	Gene-editing, RNA-based therapies
*DSP*	Desmosomal dysfunction, inflammation, fibrosis	Ventricular ectopy, inflammatory ‘hot phase’ episodes	Intermediate risk	Anti-inflammatory and anti-fibrotic approaches
*FLNC*	Z-disc and cytoskeletal disruption	Arrhythmogenic, scar-mediated phenotype	High arrhythmic risk; low end-stage HF	Genotype-specific surveillance and device strategies, molecular therapy development
*TTN*	Sarcomeric scaffolding dysfunction	HF-responsive; peripartum risk	Lower risk, not benign	GDMT optimization, emerging molecular therapies
*BAG3* ^a^	Protein quality control defects	Progressive HF phenotype	High HF risk	AAV-mediated gene replacement therapy

AAV, adeno-associated virus; DCM, dilated cardiomyopathy; GDMT, guideline-directed medical therapy; HF, heart failure; ICD, implantable cardioverter-defibrillator; MEK, mitogen-activated protein kinase; RNA, ribonucleic acid.

^a^B*AG3* was not included in the primary analysis of the index study; included here for context given emerging evidence of high-risk phenotype and active gene therapy development.

Collectively, these developments signal a shift from mechanism-agnostic treatment towards therapies informed by disease biology. All such advances depend on one prerequisite: knowledge of the genotype. Genetic testing is increasingly central to risk assessment, family screening, surveillance strategies, and eligibility for emerging genotype-directed therapies. Without a genetic diagnosis, opportunities for individualized management and participation in genotype-specific therapeutic trials may be missed. As precision therapies continue to evolve, genetic information is likely to become increasingly important in routine cardiovascular care.

Cannie and colleagues provide an important contribution to the evidence base for genotype-stratified risk prediction in DCM, confirming a clear hierarchy of adverse event rates and demonstrating that the predictors of those events differ across genetic subtypes. These findings have several important implications for clinical practice and future investigation. First, obtaining a detailed family history and maintaining a low threshold for genetic testing should be part of routine evaluation in patients with DCM. The risk hierarchy established here, and the genotype-specific therapeutic opportunities now emerging, make the case that genetic testing is increasingly relevant to clinical care. Second, risk models must integrate genotype with phenotype, imaging, and longitudinal clinical data because models built on unselected DCM populations may fail to capture the biological heterogeneity of individual genetic subtypes. Third, clinical trials for precision therapies must stratify by genotype from the outset. Addressing the limitations imposed by small genotype-specific cohorts will require coordinated international registries and genotype-enriched trial designs. Fourth, international registries require harmonized variant classification to minimize ascertainment bias and enable the genotype-specific multivariable modelling that was not possible here. As genotype-specific risk models and therapies continue to evolve, genetic information is likely to move from an adjunctive tool to a central component of precision cardiovascular medicine. Genotype is no longer merely prognostic. It is a framework for understanding disease biology and an opportunity to individualize care.
